# Insights into enhancing *Centella asiatica* organ cell biofactories via hairy root protein profiling

**DOI:** 10.3389/fpls.2023.1274767

**Published:** 2023-10-30

**Authors:** Miguel Angel Alcalde, Diego Hidalgo-Martinez, Roque Bru Martínez, Susana Sellés-Marchart, Mercedes Bonfill, Javier Palazon

**Affiliations:** ^1^ Biotechnology, Health and Education Research Group, Posgraduate School, Cesar Vallejo University, Trujillo, Peru; ^2^ Department of Biology, Healthcare and the Environment, Faculty of Pharmacy and Food Sciences, University of Barcelona, Barcelona, Spain; ^3^ Plant Proteomics and Functional Genomics Group, Department of Biochemistry and Molecular Biology, Soil Science and Agricultural Chemistry, Faculty of Science, University of Alicante, Alicante, Spain

**Keywords:** *Rhizobium rhizogenes*, biomarkers, centelloside production, molecular farming, plant biotechnology

## Abstract

Recent advancements in plant biotechnology have highlighted the potential of hairy roots as a biotechnological platform, primarily due to their rapid growth and ability to produce specialized metabolites. This study aimed to delve deeper into hairy root development in *C. asiatica* and explore the optimization of genetic transformation for enhanced bioactive compound production. Previously established hairy root lines of *C. asiatica* were categorized based on their centelloside production capacity into HIGH, MID, or LOW groups. These lines were then subjected to a meticulous label-free proteomic analysis to identify and quantify proteins. Subsequent multivariate and protein network analyses were conducted to discern proteome differences and commonalities. Additionally, the quantification of rol gene copy numbers was undertaken using qPCR, followed by gene expression measurements. From the proteomic analysis, 213 proteins were identified. Distinct proteome differences, especially between the LOW line and other lines, were observed. Key proteins related to essential processes like photosynthesis and specialized metabolism were identified. Notably, potential biomarkers, such as the Tr-type G domain-containing protein and alcohol dehydrogenase, were found in the HIGH group. The presence of ornithine cyclodeaminase in the hairy roots emerged as a significant biomarker linked with centelloside production capacity lines, indicating successful *Rhizobium*-mediated genetic transformation. However, qPCR results showed an inconsistency with rol gene expression levels, with the HIGH line displaying notably higher expression, particularly of the *rolD* gene. The study unveiled the importance of ornithine cyclodeaminase as a traceable biomarker for centelloside production capacity. The strong correlation between this biomarker and the *rolD* gene emphasizes its potential role in optimizing genetic transformation processes in *C. asiatica*.

## Introduction

*Centella asiatica* is a perennial plant, native to parts of Asia, and has garnered significant attention in modern times due to its potential health benefits and diverse applications in medicine and cosmetics (Gray et al., 2017).

The primary bioactive compounds identified in *C. asiatica* are centellosides, which are categorized as pentacyclic triterpenoid saponins. These compounds are utilized to treat a variety of conditions including skin ailments, nervous disorders, and venous insufficiency. The centelloside biosynthesis pathway originates from the mevalonate pathway, ultimately yielding farnesyl diphosphate (FPP) as a sesquiterpene precursor. Squalene synthase further converts FPP into squalene, serving as an intermediate (Gallego et al., 2014). It undergoes oxidation to form 2,3-oxidosqualene, a pivotal branching point in both sterol and triterpenoid saponin biosynthesis. This compound cyclizes into a protosteryl or dammarenyl cation, which subsequently generates various products, including the oleanyl cation (Haralampidis et al., 2002).

The oleanyl cation, catalyzed by α/β-amyrine synthase, leads to the production of α or β-amyrin (Azerad, 2016). After cyclization, further diversity in the resulting compounds is introduced through diverse modifications which are facilitated by enzymes like cytochrome P450-dependent monooxygenases and glycosyltransferases. UDP-glycosyltransferases (UGTs) play a key role in glucosylating asiatic acid and madecassic acid to yield asiaticoside and madecassoside (Kim et al., 2017).

The field of plant biotechnology has experienced significant advancements in recent years, marked by a growing interest in leveraging genetic transformation for diverse applications. In this context, hairy roots obtained by *Rhizobium*-mediated genetic transformation constitute a promising biotechnological platform, owing to their remarkable potential for specialized metabolite production and rapid growth (Gutierrez-Valdes et al., 2020).

Hairy root cultures are initiated through the random integration of a segment of *Rhizobium rhizogenes* DNA (T-DNA), mainly *rol* and *aux* genes, derived from the Ri-DNA plasmid into the plant cell genome, where the expression of the genes carried out by the T-DNA promotes rooting at the site of infection (Veena and Taylor, 2007). Concurrently with this stochastic integration process, the quantity of integrated heterologous genes presents a pertinent yet unexplored aspect. Due to the variable copy numbers of introduced transgenes, specifically T-DNA genes in this instance, have the potential to exert an influence on the collective expression levels of target genes, consequently affecting protein composition and metabolic pathways within hairy roots (Bhat and Srinivasan, 2002).

To fully exploit the advantages offered by hairy roots, a comprehensive understanding of the intricate molecular processes governing their development and metabolic capabilities is essential. One less-utilized tool for achieving this understanding is proteomics, which entails studying the complete set of proteins expressed by an organism and has revolutionized our comprehension of cellular processes and their intricate regulation. Within plant biology, proteomics has emerged as a powerful tool for unraveling the molecular mechanisms underpinning various physiological phenomena (Chen et al., 2020). Proteomics analysis may therefore shed new light on the genes associated with centelloside biosynthesis in *Centella asiatica* hairy roots, similar to observations reported in other plant species (Kim et al., 2003; Contreras et al., 2019; Chen et al., 2022).

Besides offering insights into the dynamic metabolic processes that drive hairy root development, advanced protein profiling techniques may uncover biomarkers of desirable traits. This approach therefore opens the way to achieving the production levels and developmental capacities in hairy roots necessary for their sustainable application as a biotechnological platform (Padilla et al., 2021). Furthermore, the use of omics techniques to study differentially expressed genes in *C. asiatica* hairy roots lays the groundwork for further investigation into the transcriptional regulation of centelloside content (Khan et al., 2023; Shilpha et al., 2023).

The objectives of this study were to conduct a comprehensive investigation into the potential role of protein profiling in hairy roots for the optimization and enhancement of *C. asiatica* organ cell biofactories. Understanding the relationship between transgene copy numbers and gene expression is crucial for developing strategies to improve the stability and performance of transformed lines in various biotechnological applications. Our work significantly extends the findings reported by Alcalde et al. (2022), where distinctive morphological and metabolic variations were observed among different *C. asiatica* hairy root lines, likely due to the random insertion of a limited number of genes from the T-DNA, particularly the *rol* and *aux* genes.

This study aims to provide new insights into the intricate molecular mechanisms governing hairy root development and their impact on the production of specialized metabolites, especially centelloside biosynthesis. By applying advanced protein profiling techniques, our research seeks to identify key proteins and biomarkers associated with enhanced organ cell biofactory performance. Ultimately, our goal is to contribute to the advancement of biotechnological applications by unveiling novel strategies to optimize the production of bioactive compounds through the manipulation of hairy root protein profiles.

## Materials and methods

### Plant material

The hairy root lines utilized in this study were established and morphologically characterized by Alcalde et al. (2022). To achieve this, we utilized the *Rhizobium rhizogenes* A4 strain and employed leaf segments from 2-month-old *in-vitro C. asiatica* seedlings as explants. These leaf segments, measuring 1.5–2 cm², were cut and exposed to *R. rhizogenes* colonies, then cultured at 25°C. Following this, the explants were co-cultivated in solid MS hormone-free medium with 3% sucrose and pH set at 5.8. After 48 hours of cocultivation in the dark at 28°C, the explants were transferred to fresh solid MS medium containing 500 mg/l cefotaxime. The emerging hairy roots were subsequently excised and placed on a fresh solid MS medium with 500 mg/l cefotaxime in darkness at 25°C. This process was repeated every 2 weeks for approximately 2 months to eliminate bacteria from the culture.

Transformation confirmation was conducted using a semi-quantitative RT-PCR approach. This method allowed us to detect both the integration and expression of *R. rhizogenes* T-DNA genes (*rolA, rolB, rolC*, and *aux1*) at the transcript level across the different hairy root lines. The validated lines were categorized as HIGH (4.96 ± 0.75), MID (2.48 ± 0.07), or LOW (0.54 ± 0.067), each corresponding to their respective centelloside production levels, expressed in milligrams per gram of dry weight. As a comparative control, wild adventitious (Adv) roots were excised from *in vitro C. asiatica* seedlings and cultivated on solid MS medium at 25°C in darkness.

Five samples, one gram as the initial fresh weight, from the HIGH (formerly designated as L1 by Alcalde et al. (2022), MID (L10), LOW (L3) hairy root, and adventitious root were grown on solid MS medium at 25°C in darkness and subcultured every two weeks. Sampling for protein extraction was conducted two weeks after the last subculture.

### Genomic DNA isolation

Hairy root tissue (200 mg) was pulverized in liquid nitrogen and transferred to a 1.5 mL tube. To this was added 0.75 mL of extraction buffer (50 mM EDTA, pH 8.0; 100 mM Tris, pH 8.0; and 500 mM NaCl), along with 0.6 μl of β-mercaptoethanol and 50 μl of 20% SDS. The mixture was incubated at 65°C for 10 minutes. Subsequently, 250 μl of 5 M potassium acetate was introduced, followed by an ice incubation for 20 minutes. The sample was then centrifuged at 4°C for 20 minutes at 10000 g. After recovering the supernatant, 1 mL of isopropanol was added, and the solution was kept at -20°C for 1 hour. The resulting pellet was subjected to centrifugation for 15 minutes at 10000 rpm, followed by drying.

To the dried pellet, 140 μl of T10E1 buffer (Tris 10 mM, EDTA 1 mM) was added. This mixture was then centrifuged for 10 minutes at 14000 g, the supernatant was retained, and 15 μl of 3 M sodium acetate and 100 μl of isopropanol were incorporated into the sample. After mixing, the supernatant was again recovered, and centrifugation was carried out for 10 minutes at 14000 g. The resulting pellet was dried at 37°C for 10 minutes, followed by the addition of 30 μl of T10E1 buffer. Finally, the purity of the DNA was assessed using a NanoDrop 2000 Spectrophotometer (Thermo Scientific) and 1 μl of RNAse (10 mg/mL) was introduced to remove residual RNA.

### Determination of gene copy number by qPCR

The genomic DNA (gDNA) from each sample was subjected to various dilutions, spanning concentrations from 100 ng/μl to 5 μg/μl. The dilutions were quantified utilizing a NanoDrop 2000 Spectrophotometer (Thermo Scientific). For the quantification of copy numbers of transgenes (*rol*A, *rol*B, *rol*C, and *rol*D), primer sequences were designed using Primer-BLAST (**Table 1**). As a reference gene (internal control), β-amirin synthase (β-AS) was employed, given its single-copy nature within the genome of *C. asiatica* (Kim et al., 2005).

**Table 1:** List of primers used for gene copy number estimation and gene expression.

Gene	Primer sequence from 5’ to 3’
*rolA*	FW: GAATGGCCCAGACCTTTGGA
	RV: TTGGTCAGGGAGGAAATCGC
*rolB*	FW: CAACCGGATTTGGCCAGAGA
	RV: ATAGGGTTGCATCGTGGTCG
*rolC*	FW: CGCGCTCATCACCAATCTTC
	RV: ACAGAAAGTGCGGCGAAGTA
*rolD*	FW : GCGTCGTTCCTCCCTATCAG
	RV: TCTGGCAAGATCGCCACAAA
*β-AS*	FW: CGGAGATTTCCCTCAGCAGG
	RV: CACAAGCGTTTGCGGTACTC
*β-actin*	FW: TGACAATGGAACTGGAATGG
	RV: CAACAATACTGGGGAACACT

The quantitative polymerase chain reaction (qPCR) assays were conducted using the QuantStudio3 System (Thermo Fisher). Amplifications were carried out in 10 μl reaction solutions, comprising 1 μl of gDNA from each dilution sample, 2 μl of sterile milliQ H2O, 5 μl of iTaq™ Universal SYBR^®^ Green Supermix (BIO-RAD), and 1 μl of each specific primer at a concentration of 10 μM. The PCR conditions consisted of an initial step at 95°C for 60 seconds, followed by 40 cycles of denaturation at 95°C for 10 s, annealing at 60°C for 20 s and extension at 72°C for 30 s. The specificity of each primer pair was validated by melting curve analysis (95°C for 15 s, a temperature range of 60–95°C with a ramp rate of 0.1°C/s, followed by 95°C for 15 s). To ensure reproducibility, each assay was performed with three technical replicates for each of the three biological samples.

To calculate the transgene copy number, we adopted the formula outlined by Kanwar et al. (2022) X/R= 10^(((Cx-Ix)/Sx-(Cr-Ir)/Sr)), incorporating the slope and intercept values obtained from the standard curve. The average Ct values obtained from the four dilutions were utilized. These collected values were then integrated into an equation, which was subsequently plotted. In the context of each group of hairy root lines (HIGH, MID, and LOW), Cx and Cr represent the average Ct values corresponding to the transgene and β-AS, respectively. Ix and Ir denote the intercepts associated with the transgene and β-AS, while Sx and Sr signify the slopes for the transgene and β-AS, respectively. To derive the copy number, the X/R value is multiplied by two.

### Gene expression

The *rol* genes expression in the transgenic lines was verified using quantitative real-time polymerase chain reaction (qRT-PCR). Gene normalization was accomplished using the β-actin gene. Total RNA was isolated from plant material utilizing TRIzol reagent (Invitrogen, Carlsbad, CA). For the qRT-PCR, cDNA was synthesized from RNA treated with DNase I (Invitrogen, Carlsbad, CA) using SuperScript IV reverse transcriptase (Invitrogen, Carlsbad, CA) according to the manufacturer’s instructions. The qRT-PCR assays were performed employing the iTAqTM Universal SYBR Green Supermix (Bio-Rad, Hercules, CA, USA) in the QuantStudio3 System (Thermo Fisher). Each sample was analyzed in triplicate under the following conditions: an initial step at 95°C for 60 s, followed by 40 cycles of denaturation at 95°C for 10 s, annealing at 60°C for 20 s, and extension at 72°C for 30 s. Subsequent to amplification, a melting curve analysis was conducted. To check reproducibility, each assay was performed with technical triplicates for each of the three biological samples. Gene-specific primers were designed using Primer-BLAST (**Table 1**).

### Label-free proteomic analysis

A time series proteomic experiment was conducted using quadruplicates of whole cell extracts from each transgenic line and adventitious roots. Trypsin protein digestion and peptide cleanup were carried out following the procedure described by Wang et al. (2006). For analysis, 30 mg of desalted peptide digests were directly injected onto a reverse phase Agilent AdvanceBio Peptide mapping column (2.1 mm x 250 mm, 2.7 μm particle size) attached to an Agilent 1290 Infinity UHPLC, coupled through an Agilent Jet Stream^®^ interface to an Agilent 6550 iFunnel Q-TOF mass spectrometer (Agilent Technologies) system.

Peptide separation was performed at 50°C using a 140-minute linear gradient of 3-40% ACN in 0.1% formic acid at a flow rate of 0.400 mL/min. Source parameters included a gas temperature of 250°C, drying gas at 14 L/min, nebulizer at 35 psi, sheath gas temperature at 250°C, sheath gas flow at 11 L/min, capillary voltage at 3,500 V, and fragmentor at 360 V. Data were acquired in positive-ion mode using Agilent MassHunter Workstation Software, LC/MS Data Acquisition B.08.00 (Build 8.00.8058.0). Operating in high sensitivity mode, MS and MS/MS data were collected in Auto MS/MS mode. This involved selecting the 20 most intense parent ions (charge states from 2 to 5) within the 300 to 1,700 m/z mass range, provided they exceeded a threshold of 1,000 counts, for subsequent MS/MS analysis. MS/MS spectra spanning the 50-1,700 m/z range were gathered with the quadrupole set to “narrow” resolution. Data acquisition was continued until either a total count of 25,000 was reached or a maximum accumulation time of 333 ms was achieved.

Each MS/MS spectrum was subjected to preprocessing using the extraction tool within the Spectrum Mill Proteomics Workbench (Agilent). This step aimed to generate a peak list and enhance spectral quality by merging MS/MS spectra sharing the same precursor (with a Δm/z < 1.4 Da and chromatographic Δt < 15s). The resulting refined dataset was then subjected to a search against the proteome database, encompassing primary species from the *Apiaceae* family and the *Rhizobium*/*Agrobacterium* genus. Additionally, the analysis included identification of contaminant proteins using the identity mode of the MS/MS search tool in the Spectrum Mill Proteomics Workbench, configured as follows: trypsin enzyme specificity, allowance for up to 2 missed cleavages, fixed modification of Cys by carbamidomethylation, variable modification of Met by oxidation, and mass tolerance of 20 ppm for precursor ions and 50 ppm for product ions. The peptide hits obtained were subjected to filtering, retaining those with a score of ≥ 6 and a percent scored peak intensity (%SPI) of ≥ 60.

The LC-MS raw files were imported into Progenesis QI for Proteomics (Nonlinear Dynamics) version 4.0, a label-free analysis software. Quantification was based on MS1 intensity. The data file with the highest number of features (peaks) served as a reference for aligning the retention times of all other chromatographic runs and for normalizing MS feature signal intensity (peak area). To address experimental variations, a robust distribution of all ratios (log(ratio)) was computed for correction purposes. MS features were filtered to encompass only those with a charge state ranging from two to five. Employing a “between subjects” experimental design mode, samples were clustered according to their respective experimental groups (Adv, LOW, MID, and HIGH). Average intensity ratios of matched features across experimental groups, along with p-values from one-way ANOVA, were automatically computed.

For protein identification, the filtered SpectrumMill peptide hits files were introduced into Progenesis QIp. Here, conflicts in peptide assignments were resolved, either by selecting the highest score as the winner or by retaining unresolved conflicts in cases of equal scores and sequences. The inferred protein list was then filtered to include entries with a score of ≥15. To determine protein abundance, the Hi-3 method described by Silva et al. (2006) and implemented in Progenesis QI for Proteomics was employed. Differential protein abundance across experimental groups was evaluated using advanced statistical tools available in Progenesis QIp, including ANOVA and Power analysis.

### Statistical analysis and protein network

The protein abundance datasets were imported into SIMCA-P software, version 14.1 (Umetrics, Umea, Sweden) for further analysis. To ensure comparability, all variables underwent pareto scaling prior to multivariate analysis. The pareto-scaled variables underwent orthogonal partial least squares discriminant analysis (OPLS-DA) to elucidate distinctive components within the sample set. The OPLS-DA predictive component loading was visualized using S-plots, a technique proven effective for enhancing model interpretation and biomarker discovery (Wiklund et al., 2008). Model quality assessment was performed using R2X (cumulative) and Q2 values, following the criteria established by Triba et al. (2015).

Statistical analyses to assess differences in protein levels were conducted using GraphPad Prism 7.0 software. Analysis of variance (ANOVA) was employed, followed by Tukey’s *post hoc* test to determine significant variations between protein levels. To discern pairwise differences between means, the Tukey-Kramer multiple-comparison test was utilized, with a significance threshold set at p < 0.05. To predict protein-protein interactions, a list of protein identifiers was submitted to the web interface of the STRING. (2023). https://string-db.org/ [Accessed July 27, 2023].

## Results

### Proteomic profiles in hairy and adventitious roots

The experimental design proposed in this study aimed to investigate and compare the complete set of soluble proteins expressed in both transformed and adventitious roots. The Adv roots were collected from *in vitro*-grown *C. asiatica* seedlings. The transformed roots, which were morphologically and phytochemically characterized by (Alcalde et al., 2022), were categorized as HIGH, MID, or LOW based on their respective capacities for the production of centellosides – the key bioactive compounds of *C. asiatica* plants.

Samples of each root group were subjected in quadruplicate to a label-free proteomic analysis, which was conducted by searching against the Uniprot databases for *Apiaceae* taxonomy (see Material and Methods). A total of 213 quantifiable proteins were identified and selected based on specific criteria: they were required to have a SCORE ≥15, p-value ≥ 0.05, and a fold change (FC) ≥2.

**Figure 1:**
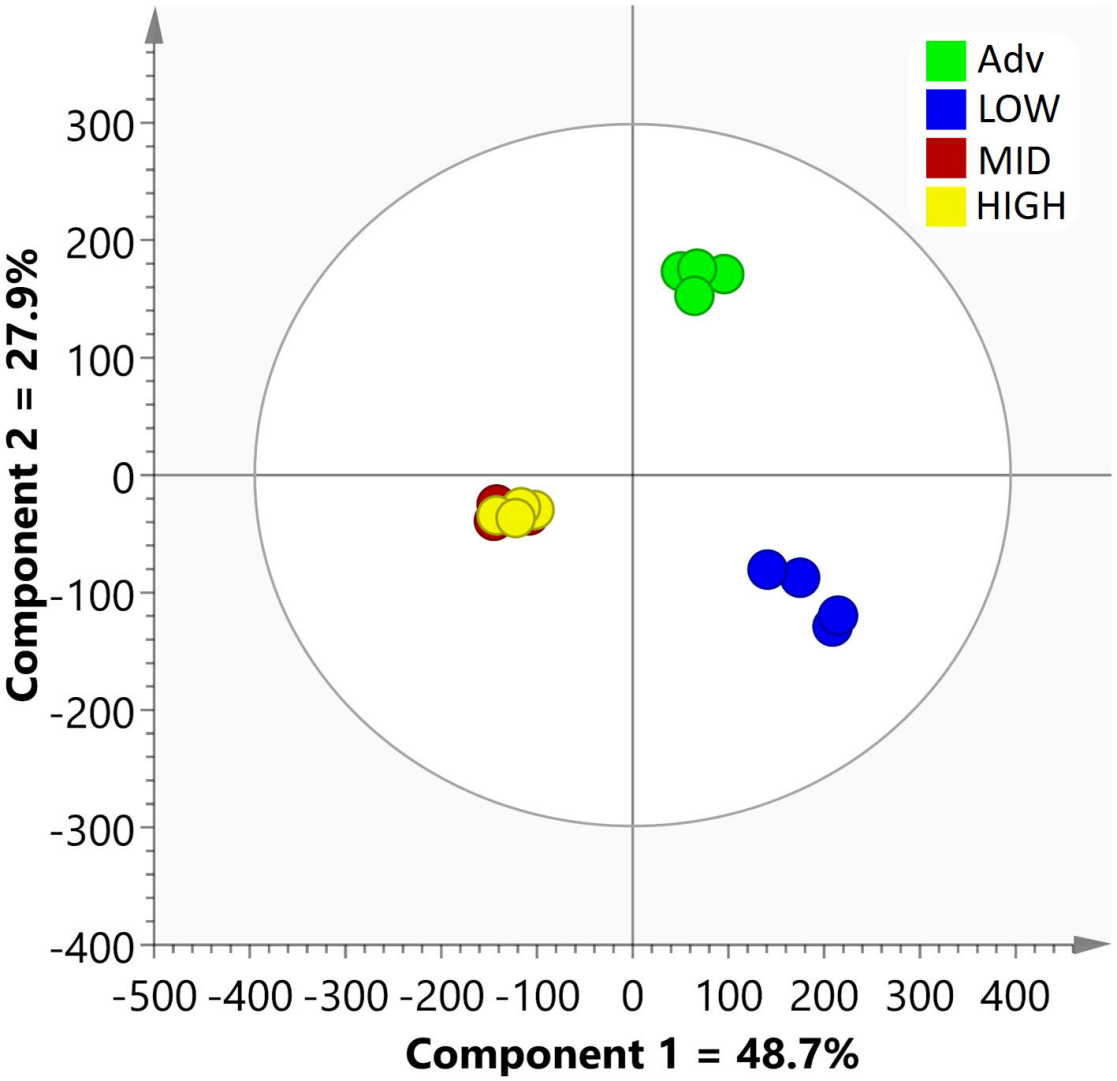
Score scatter plot of the OPLS-DA model conducted using the quantified data of 213 proteins from the Apiaceae database for the various root lines.

As a first approximation after this data filtering, an OPLS-DA was carried out with the 213 proteins quantified with the *Apiaceae* database. The score scatter plot of this model (**Figure 1**) displays the variation between the groups, with components 1 (48.7%) and 2 (27.9%) representing the maximum separation. The model provided a satisfactory explanation of the variation, showing a good fit with an R2X (cum) value of 0.786. Further, the reliability of the model was confirmed by cross-validation, resulting in a Q2 (cum) value of 0.861. Interestingly, the proteomes of HIGH and MID roots exhibited minimal differences, whereas both differed significantly from the ADV and LOW proteomes. Notably, the most pronounced difference was observed between the LOW group and the others.

The list of quantified plant proteins was analyzed using STRING. Out of 213 proteins, 184 were found in the STRING database and utilized to construct a network, displaying significant interactions (refer to **Supplementary Figure 1**). The identified proteins were further classified based on their biological processes (Gene Ontology) and KEGG Pathways (see **Supplementary Table 1**). The classification revealed the recurrence of proteins associated with essential processes such as photosynthesis and amino acid biosynthesis. Additionally, proteins related to pathways of specialized metabolites, including phenylpropanoids, were also prominent.

**Figure 2:**
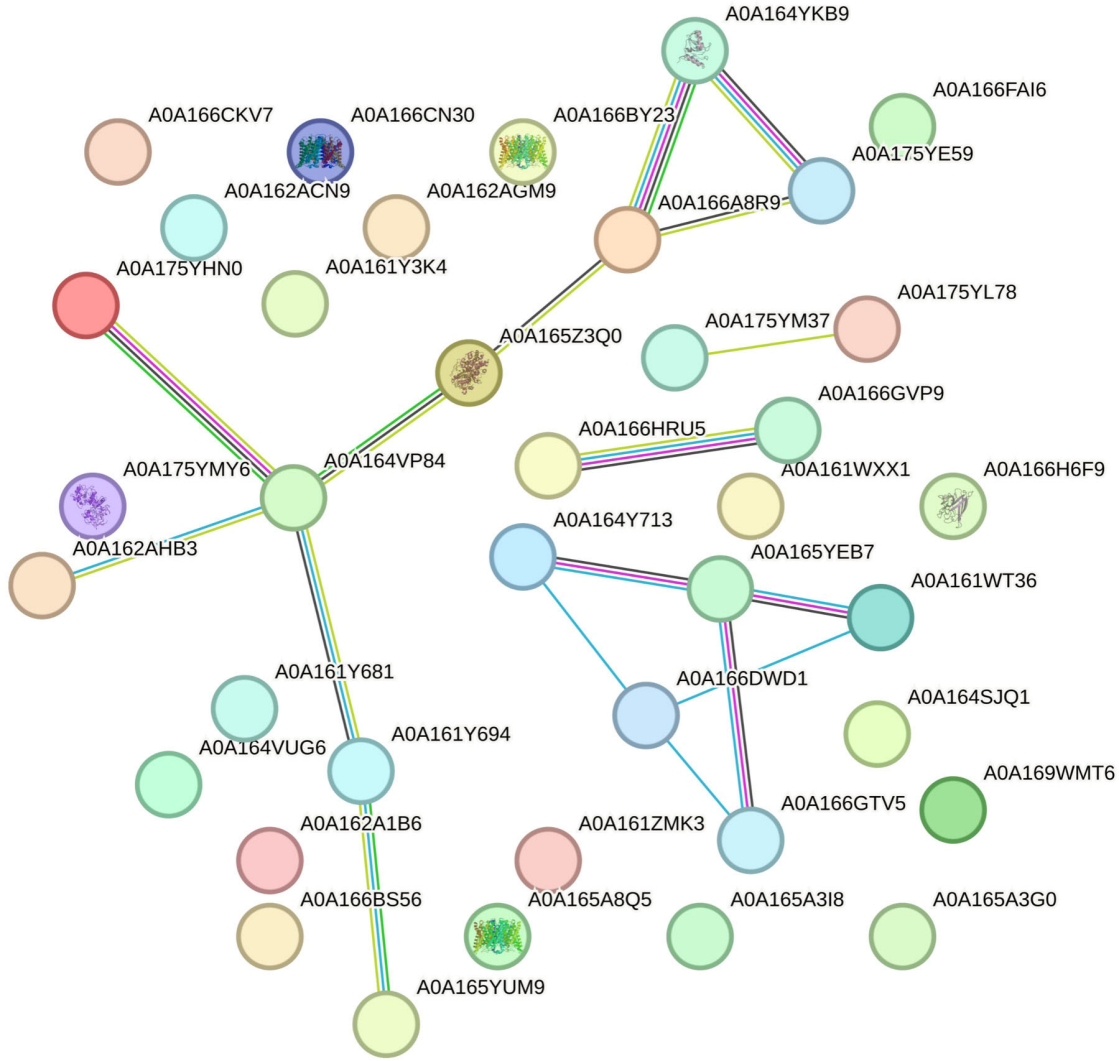
Protein network constructed using the STRING web interface, illustrating the network formed by 38 proteins found in the STRING database. Network nodes depict proteins, and edges symbolize protein-protein associations. The accompanying legend displays the UniProt accession numbers.

In the next step, ANOVA and Tukey tests were conducted to identify proteins with significant differences among the different root lines. The comparison between the HIGH and LOW lines revealed that only 44 proteins exhibited statistical variations (**Supplementary Table 2**). Out of these, only 38 could be utilized to construct a protein network using STRING (**Figure 2**). Surprisingly, the majority of the differentially expressed proteins were found in higher abundance in the LOW hairy roots, and only a few were found in greater quantities in the HIGH group. The differentially expressed proteins are as follows: alcohol dehydrogenase, UDP-arabinopyranose mutase, Tr-type G domain-containing protein, plant heme peroxidase, D-3-phosphoglycerate dehydrogenase, and ketol-acid reductoisomerase.

The proteins from this network were further classified based on their biological processes and KEGG pathways (**Supplementary Table 3**). This classification revealed the absence of proteins associated with photosynthesis, previously found when the Adv roots were included in the multivariable analysis. Additionally, proteins related to pantothenate, CoA, and phenylpropanoid and amino acid biosynthesis received good scores in the classification.

### Biomarker discovery

**Figure 3:**
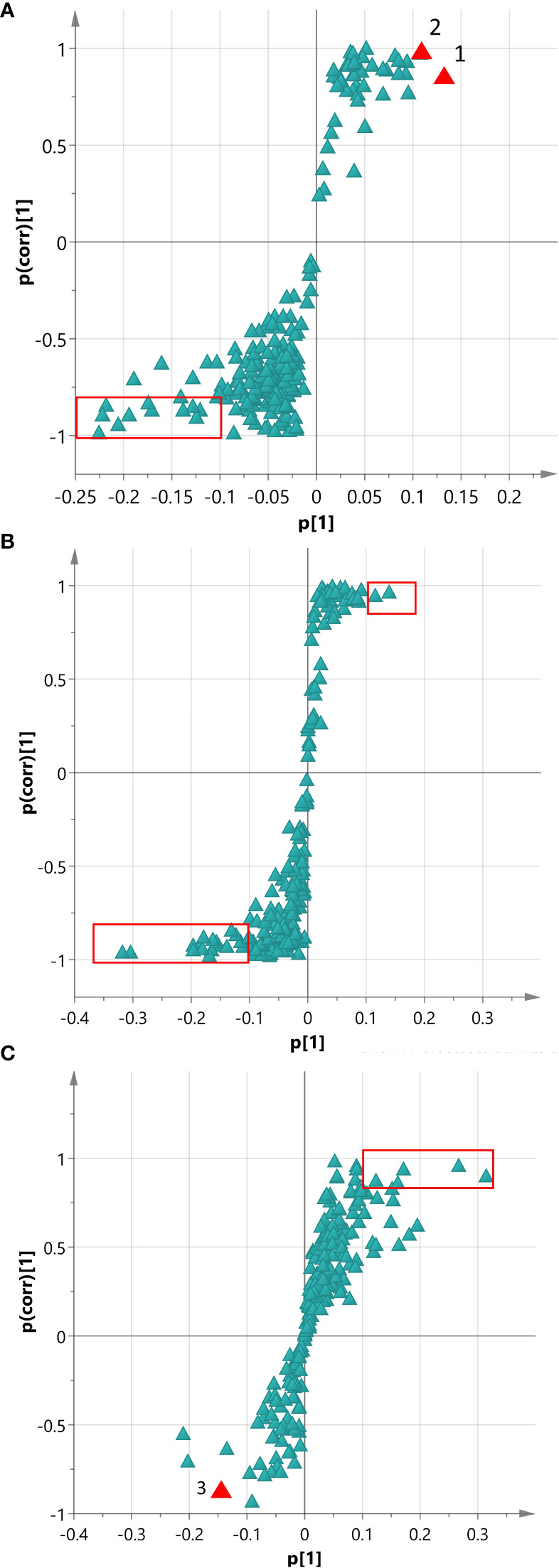
The S-plot depicts the magnitude (modeled covariation) and reliability (modeled correlation) of each protein, represented by triangles. The red rectangle highlights the region with high magnitude (≥ |0.1|) and reliability (≥ |0.8|). **(A)** S-plot analysis of the Adv vs HIGH OPLS-DA model: Number 1 corresponds to Tr-type G domain-containing protein, and number 2 corresponds to alcohol dehydrogenase protein. **(B)** S-plot analysis of the LOW vs HIGH OPLS-DA model. **(C)** S-plot analysis of the MID vs HIGH OPLS-DA model: Number 3 represents Bet v I/Major latex protein.

To identify significant markers among the Adv and hairy root groups, an OPLS-DA analysis was conducted for each comparison using the 213-protein dataset mentioned above. To aid the visualization of the discrimination model in terms of biomarkers, an S-plot (Wiklund et al., 2008) was utilized to filter potential proteins. The S-plot of the Adv vs HIGH model (**Figure 3A**) illustrates the magnitude (modeled covariation) and reliability (modeled correlation) of each protein. Putative biomarkers were identified based on a small set of proteins exhibiting high magnitude (≥ |0.1|) and reliability (≥ |0.8|). In this specific model, we identified only two biomarkers positively correlated with the HIGH group: a Tr-type G domain-containing protein and an alcohol dehydrogenase protein. In contrast, 13 proteins were correlated with the Adv group (**Supplementary Table 4**), most of them related to photosynthesis.

The S-plot analysis of the Adv vs MID model identified alcohol dehydrogenase protein as a putative biomarker, showing a significant correlation with the MID group. Intriguingly, the clearest distinction between two groups was observed in the Adv vs LOW model. At least six distinct proteins were found to be positively correlated with the LOW roots, including the PCMH-type FAD-binding domain, eukaryotic translation initiation factor, peroxidase, and cysteine protease.

Additional comparisons between the transgenic lines revealed that Tr-type G domain-containing protein was the only one that could potentially serve as a reliable indicator to distinguish between the HIGH/MID and the LOW lines (**Figure 3B**). A detachable potential biomarker differentiating between HIGH and MID was the Bet v I/Major latex protein, which was more strongly correlated with the MID group (**Figure 3C**).

Finally, we conducted a proteomic analysis by searching against the Uniprot databases for *Rhizobium/Agrobacterium* taxonomy, as described in the Material and Methods section. A total of 100 quantifiable proteins were identified and carefully selected based on the specific criteria described above. This dataset was then used for an OPLS-DA analysis comparing Adv and HIGH lines, followed by an S-plot to identify potential biomarkers. The proteins showing high magnitude (≥ |0.1|) and reliability (≥ |0.8|) were manually curated to ensure that only proteins potentially originating from *Rhizobium* via T-DNA were included, filtering out any proteins that could be derived from other sources, to ensure the selection of true *Rhizobium* biomarkers. Consequently, only the ornithine cyclodeaminase protein (OCD) complied with the curation process.

In *R. rhizogenes*, OCD is located in the T-DNA region of the Ri plasmid and is referred to as RolD by Trovato et al. (2001). The presence of OCD in the hairy roots is noteworthy, as this enzyme plays a crucial role in synthesizing proline from ornithine in a single step. According to Trovato et al. (2018), this metabolic capability could be functionally involved in the process of root elongation and/or maturation. The production of higher amounts of proline, an important osmolyte and signaling molecule, may contribute to stress tolerance and growth regulation in the hairy roots, making it a significant biomarker of *Rhizobium* -mediated genetic transformation.

### Impact of transgene copy number on gene expression

Based on the discovery of the OCD biomarker, and with the objective of investigating the potential impact of the transgenes from the T-DNA of *R. rhizogenes* on the hairy root proteome profiles, we proceeded to quantify the number of copies and the level of expression of the *rol* genes in the transgenic lines.

**Figure 4:**
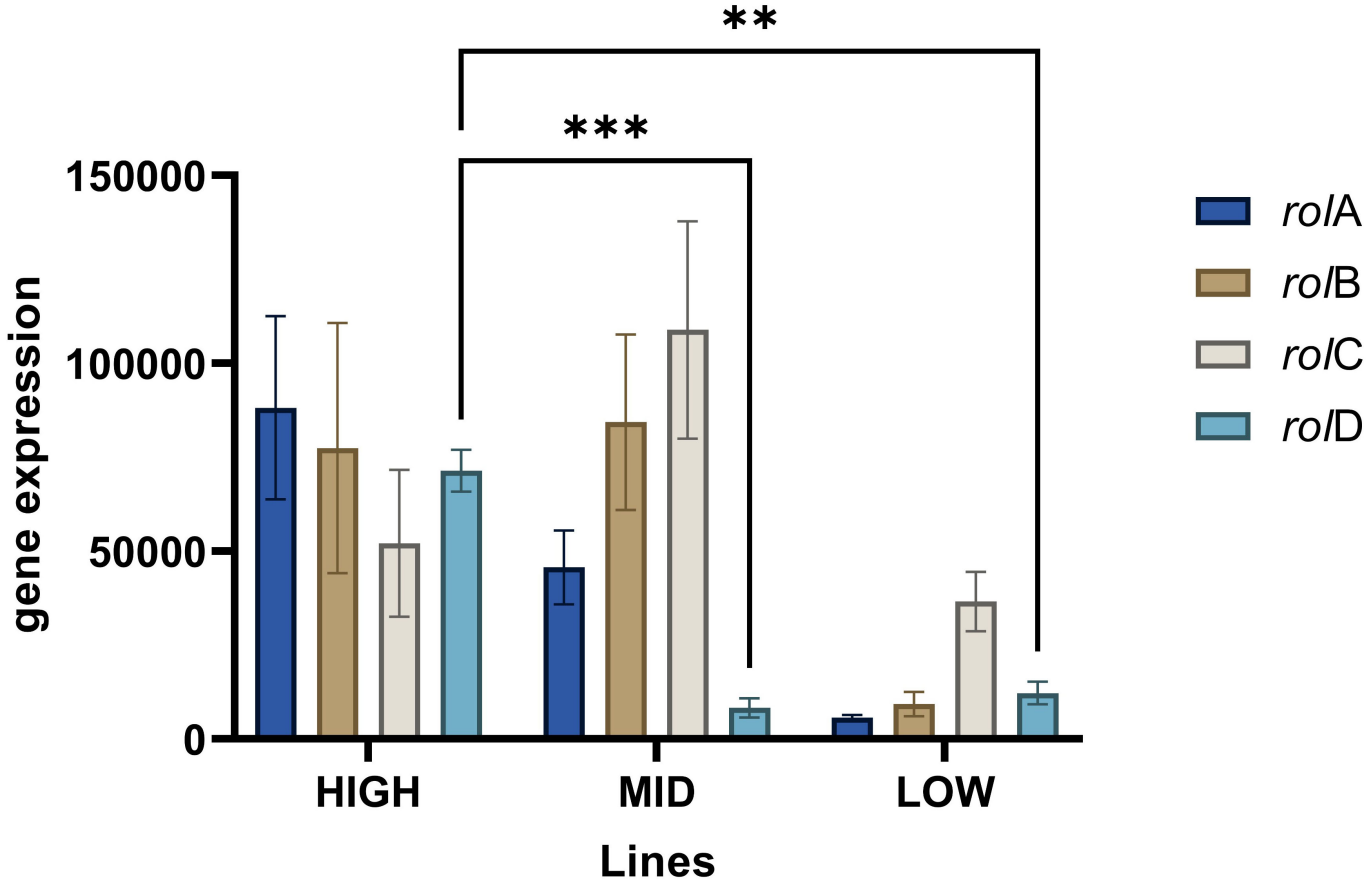
Normalized gene expression values from the transgenic lines: HIGH, MID, and LOW. Asterisks indicate statistical differences among the lines solely for the rolD gene (a = 0.05). Data represent the mean ± SD of three replicates.

The transgene copy number for all transgenic lines was determined using qPCR relative to the endogenous reference gene β-amyrin synthase (β-AS), a gene involved in the biosynthesis of centellosides (Kim et al., 2005). The quantifiable parameters collected are shown in **Supplementary Table 5**. After calculations, all the tested lines were estimated to have two copies of each transgene (see **Table 2**). Nevertheless, upon scrutinizing the gene expression patterns, clear distinctions emerged between the groups (**Figure 4**). In particular, the HIGH line stands out due to heightened expression levels of most *rol* genes, especially the *rol*D gene, which was found to be 5 to 8 times more pronounced than in the LOW and MID lines, respectively. These differences suggest that the presence of two copies of the transgenes does not necessarily correlate with uniform expression levels. Furthermore, the greater abundance of *rol*D gene transcripts appears to correlate with the OCD biomarker, suggesting its potential significance.

**Table 2:** Estimated number of copies of each transgene.

gene	Line	2(X/R)	Estimated number of copies
*rolA*	HIGH	2.001	2
	MID	2.248	2
	LOW	1.417	1-2
*rolB*	HIGH	1.325	1-2
	MID	1.999	2
	LOW	2.000	2
*rolC*	HIGH	2.000	2
	MID	2.248	2
	LOW	2.238	2
*rolD*	HIGH	2.000	2
	MID	1.997	2
	LOW	2.000	2

## Discussion

The literature contains numerous examples of plant biofactories based on hairy root cultures designed to produce plant bioactive compounds scarcely synthesized in nature (Hasnain et al., 2022; Bapat et al., 2023; Sonkar et al., 2023). Most of these studies adopt empirical approaches, concentrating on establishing hairy root cultures to explore their production of specialized plant compounds and optimizing the biotechnological production system. In contrast, only a limited body of research has taken a rational approach, trying to understand how a specific set of genes from *R. rhizogenes* (*rol* genes) can modify cell growth and metabolism (Mauro et al., 2017; Sarkar et al., 2018).

With the aim of increasing our understanding of the effects of *rol* genes on plant metabolism, in the present study, an experimental design was meticulously crafted to determine a complete representative repertoire of soluble proteins expressed within two distinct root types: transformed (carrying the *rol* genes) and adventitious roots (excised directly from the plants and cultured separately). The protein profiling approach employed in this study not only provides insights into the dynamic metabolic processes underpinning hairy root development but also furnishes a lens through which we can discern biomarkers associated with traits that bestow the coveted production and developmental capacities required for a sustainable biotechnological platform.

The Adv roots were sampled from *C. asiatica* seedlings cultivated within a carefully controlled *in vitro* environment. Moreover, strong precautions were exercised to regulate the growth conditions of these roots, including light shielding, to ensure a robust basis for comparison with the conventionally grown transformed roots. The specific transformed lines were selected according to their previous characterization by (Alcalde et al., 2022), which benchmarked their performance for biotechnological production application.

Despite the care taken in cultivating the Adv roots, proteins related to photosynthesis were surprisingly evident in their protein profiles, the levels being significantly higher than in the transgenic roots. This discrepancy led us to investigate further and prompted a comparison between the HIGH, MID, and LOW lines. The results of this analysis proved to be particularly relevant in our quest to unravel the protein networks that significantly impact the performance of hairy root lines in biotechnological applications. The comparisons revealed several key biomarkers, notably the Tr-type G domain-containing protein, alcohol dehydrogenase protein, and Bet v I/Major latex protein.

The Tr-type G domain-containing protein is categorized within the GTPase family of classical translation factors under the EF-G/EF-2 subfamily. These elongation factors play a fundamental role in the process of translation, which constitutes a fundamental step in the intricate process of protein synthesis (Xu et al., 2022). This observation aligns well with the morphological traits characterizing the HIGH line, as reported by (Alcalde et al., 2022), which demonstrated superior elongation, branching, and biomass production.

Alcohol dehydrogenases (ADH) in plants, pioneering subjects in early molecular research (App and Meiss, 1958), play an important role in orchestrating the conversion of ethanol to acetaldehyde, as described by Strommer (2011). Notably, increased ADH expression in *Arabidopsis* has been associated with enhanced tolerance to anoxia and improved root growth, as demonstrated by Shiao et al. (2002). Interestingly, these traits align with the morphological characteristics of the hairy roots in our study.

The major latex protein (MLP) subfamily has pivotal functions in defense and stress responses, and forms the second-largest category of the birch pollen allergen Bet v 1 superfamily, as elucidated by Yuan et al. (2020). MLPs are frequently sequestered within laticifers – latex-filled tubular structures – distributed throughout the plant. Such compartments serve as optimal reservoirs for defense metabolites, functioning as a frontline defense mechanism, an aspect highlighted by Musidlak et al. (2020).

MLPs also play a role in enhancing stress tolerance through intricate plant hormone signaling pathways. It is plausible that MLPs interact with indole-3-acetic acid (IAA) and participate in the auxin signaling pathway, influencing the IAA levels in hairy roots. This is significant due to the relevant role that this plant hormone plays in root induction and development (Patten and Glick, 2002; Duca and Glick, 2020). This intriguing connection could potentially explain their function as biomarkers for MID lines, as suggested by Fujita and Inui (2021).

Although numerous authors have suggested that the *rol*D gene of the *R. rhizogenes* T-DNA does not play a significant role in hairy root induction, our results contradict this hypothesis (Trovato et al., 1997; Bulgakov, 2008). The detection of the OCD biomarker indicates a potential connection between *Rhizobium*-mediated genetic alteration and the proteome composition of hairy roots. The enzymatic transformation of ornithine into proline through the OCD-like function of rolD may provide a credible rationale for its involvement in the generation of hairy roots (Trovato et al., 2008; Trovato et al., 2018). It is worth noting that prior studies have reported a significant escalation in proline levels within the growth region of primary maize roots under conditions of limited water availability, implying a crucial function of proline synthesis in maintaining root growth (Verslues and Sharp, 1999). An elevated concentration of proline has the potential to influence the synthesis of hydroxyproline-rich glycoproteins (HRGPs), encompassing extensins and arabinogalactan proteins, which serve as integral structural constituents of the plant cell wall (Okumoto et al., 2015). HRGPs are thought to oversee essential processes such as cell division, the self-assembly of the cell wall, and cell elongation, which could contribute to the noted impacts of RolD on root growth (Trovato et al., 2001). Alternatively, the promotion of root growth by *rolD* could also be associated with the reduction of ornithine, thus impacting the polyamine reservoir, where ornithine operates as a precursor. The overexpression of arginine decarboxylase, another polyamine precursor, has been demonstrated to increase putrescine levels and hinder root growth in tobacco plants (Masgrau et al., 1997). Further research into the specific role of OCD protein in root development and stress responses could enhance our knowledge of the mechanisms underlying hairy root formation. This would potentially open new avenues for biotechnological applications in agriculture and plant biotechnology, specifically in the development of new plant biofactories for the production of high added value compounds synthesized in plant roots.

The non-identification of proteins associated with the T-DNA is not unexpected and can be attributed to the particular technique and parameters employed (see Material and Methods), which might not capture the full range of proteins, especially those of lower abundance. Moreover, the observed variations in gene expression across the different lines could potentially arise from a multitude of factors, including the exact insertion site of the transgene within the genome, given that the insertion of the T-DNA is a random process (Gelvin, 2017; Singer, 2018). Our findings regarding the quantity of copies originating from the T-DNA support the notion that it is the insertion site, rather than the number of transgene copies, which exerts a more pronounced influence on elevated expression levels and subsequent protein translation. This stands in contrast to the conventional belief that comprehending the impact of transgene copy numbers is paramount for optimizing genetic transformation, thereby ensuring consistent and predictable outcomes in hairy root growth and the production of secondary metabolites (Ludwig-Müller et al., 2014). Furthermore, it should be noted that high-copy number transgenes may be more susceptible to instability, potentially resulting in the loss of the inserted genes. Therefore, gaining a comprehensive understanding of copy number dynamics remains critical for maintaining stable genetic modifications (Yang et al., 2005).

Furthermore, the intricate network of molecular mechanisms orchestrating gene expression further contributes to this variability. It is noteworthy that the interplay between transcriptional regulation, post-transcriptional modifications, and protein turnover can often lead to discrepancies between gene expression levels and actual protein abundances. This divergence reflects the complexity inherent in translating genetic information into functional proteins and shows that the underlying processes can only be comprehensively understood through a holistic approach encompassing both transcriptomic and proteomic analyses (Kumar et al., 2016; Stenton et al., 2020; Veenstra, 2021).

The findings in this work shed light on the proteomic differences among the Adv, MID, and LOW root lines, contributing to a deeper understanding of the molecular basis underlying their diverse characteristics. The comprehensive proteomic analysis performed provides valuable insights into the proteins associated with *Rhizobium* infection. The identified biomarkers hold great promise for further investigations into the mechanisms of *Rhizobium*-mediated genetic transformation and their implications in biotechnology and plant genetic engineering. Our findings underscore the importance of not only quantifying transgene copy numbers but also assessing their impact on gene expression and protein accumulation. Understanding these effects will be crucial for optimizing genetic transformation strategies and ensuring consistent and predictable outcomes in biotechnological applications.

## Data availability statement

The mass spectrometry proteomics data presented in the study have deposited to the ProteomeXchange Consortium via the PRIDE partner repository, accession number PXD045705.

## Author contributions

MA: Conceptualization, Formal Analysis, Investigation, Methodology, Software, Supervision, Writing – original draft, Writing – review & editing. DH-M: Conceptualization, Methodology, Software, Supervision, Writing – original draft, Writing – review & editing. RB-M: Methodology, Resources, Writing – original draft. SS-M: Methodology, Resources, Writing – original draft. MB: Formal Analysis, Investigation, Supervision, Writing – original draft. JP: Conceptualization, Formal Analysis, Funding acquisition, Investigation, Methodology, Supervision, Writing – original draft.
