# Supplementary material for: Insights into enhancing Centella asiatica organ cell biofactories via hairy root protein profiling
Source: Front Plant Sci. 2023 Oct 30;14:1274767. doi: 10.3389/fpls.2023.1274767 (PMC10642384; doi:10.3389/fpls.2023.1274767)
Supplement: Supplementary file 1 [file DataSheet_1.docx]

**SUPPLEMENTARY MATERIAL**

**Insights into enhancing *Centella asiatica* organ cell biofactories via hairy root protein profiling**

**Miguel Angel Alcalde^1,3^*, Diego Hidalgo-Martinez^1^, Roque Bru-Martínez^2^, Susana Sellés-Marchart^2^, Mercedes Bonfill^1^, Javier Palazon^1,^***

^1^Department of Biology, Healthcare and the Environment. Faculty of Pharmacy and Food Sciences. University of Barcelona. Spain

^2^Plant Proteomics and Functional Genomics Group, Department of Agrochemistry and Biochemistry, Faculty of Science, University of Alicante, Alicante, Spain

^3^Biotechnology, Health and Education Research Group, Posgraduate School, Cesar Vallejo University, Trujillo, Peru.

*** Correspondence:**Javier Palazon and Miguel Angel Alcalde
javierpalazon@ub.edu; miguel.psr.94@gmail.com


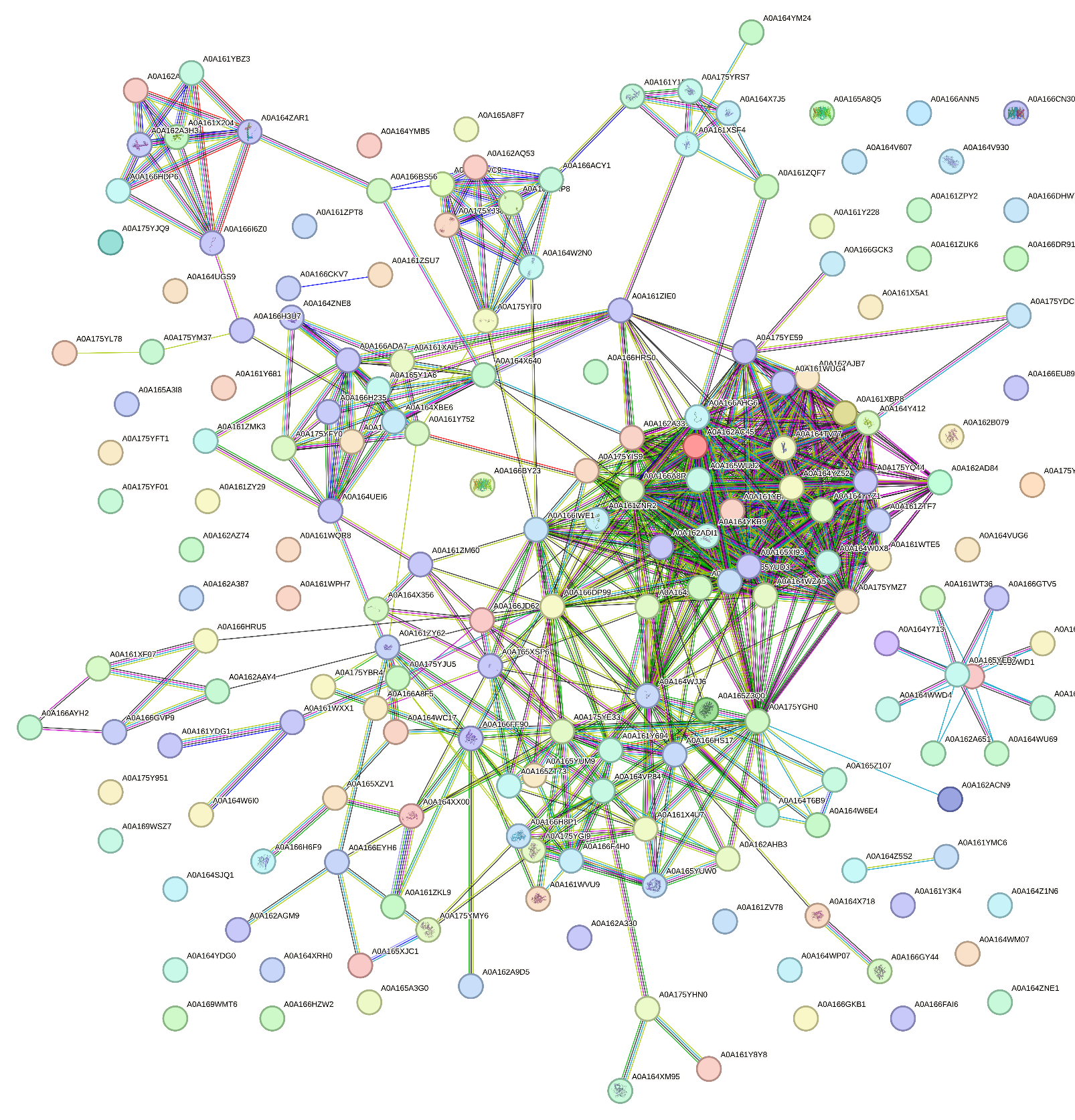


| number of nodes: | 184 |
| --- | --- |
| number of edges: | 689 |
| average node degree: | 7.49 |
| avg. local clustering coefficient: | 0.433 |
| expected number of edges: | 459 |
| PPI enrichment p-value: | < 1.0e-16 |

**Figure S1**. Protein network constructed using the STRING web interface, illustrating the network formed by 184 proteins found in the STRING database. Network nodes depict proteins, and edges symbolize protein-protein associations. The accompanying legend displays the UniProt accession numbers.


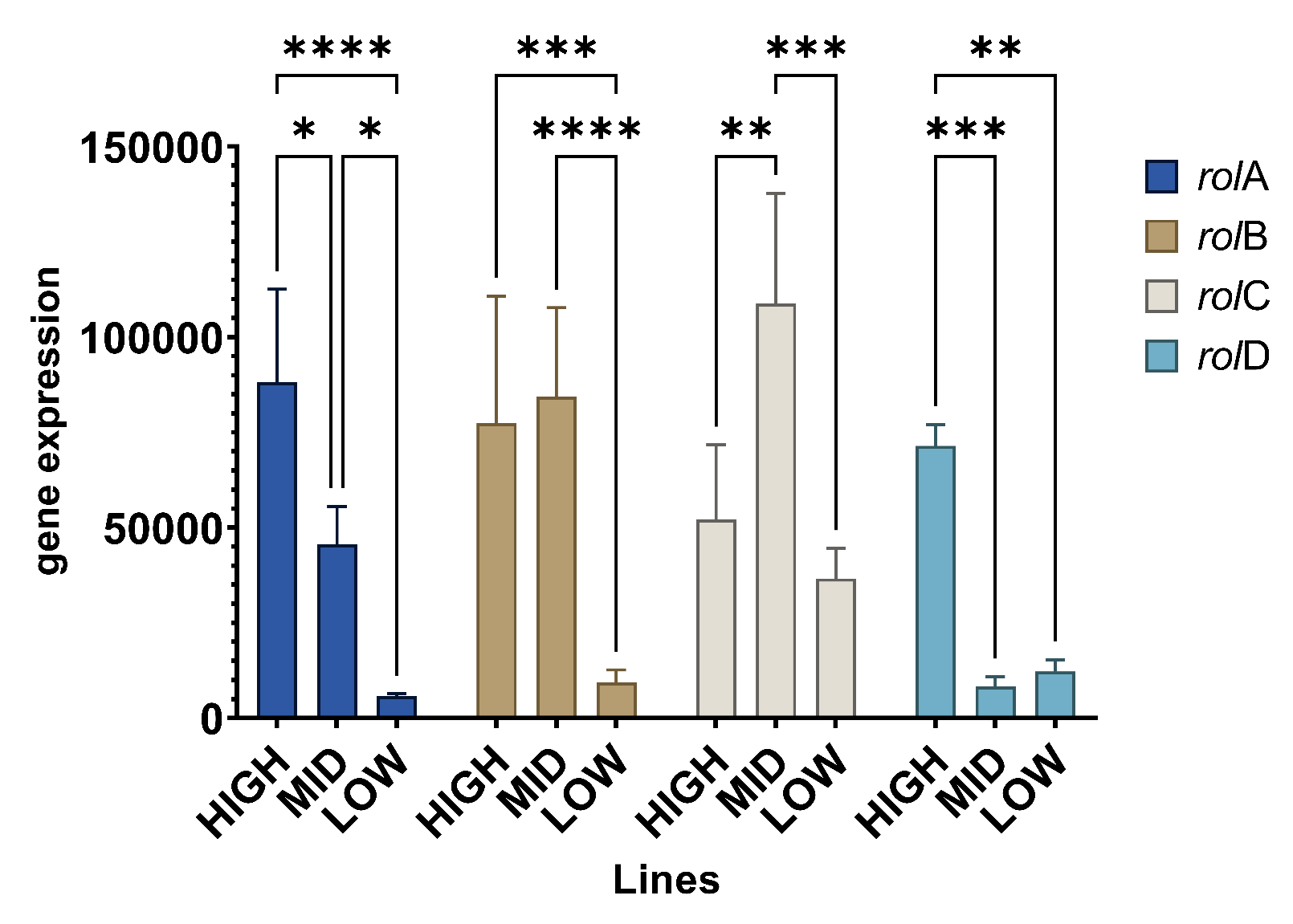


Figure S2. Normalized gene expression values from the transgenic lines: HIGH, MID, and LOW. Asterisks indicate statistical differences among the lines solely for the *rol*D gene (α = 0.05). Data represent the mean ± SD of three replicates.
